# High expression of XPA confers poor prognosis for nasopharyngeal carcinoma patients treated with platinum-based chemoradiotherapy

**DOI:** 10.18632/oncotarget.4424

**Published:** 2015-06-10

**Authors:** Xiang Fu, Jiali Hu, Hong-yu Han, Yi-jun Hua, Ling Zhou, Wen-di Shuai, Wu-ying Du, Chun-mei Kuang, Shuai Chen, Wenlin Huang, Ran-yi Liu

**Affiliations:** ^1^ Sun Yat-Sen University Cancer Center, State Key Laboratory of Oncology in South China, Collaborative Innovation Center of Cancer Medicine, Guangzhou, China; ^2^ The Eastern Hospital of the First Affiliated Hospital, Sun Yat-Sen University, Guangzhou, China; ^3^ Guangdong Provincial Key Laboratory of Tumor targeted Drugs and Guangzhou Enterprise Key Laboratory of Gene Medicine, Guangzhou Doublle Bioproducts Co. Ltd., Guangzhou, China

**Keywords:** nasopharyngeal carcinoma, xeroderma pigmentosum complementation group-A, platinum resistance, prognostic markers, chemoradiotherapy

## Abstract

In this study, we tried to explore if xeroderma pigmentosum complementation group-A (XPA) expression is likely a prognostic prediction factor for locally advanced nasopharyngeal carcinoma (NPC) patients treated with platinum-based chemoradiotherapy, which was considered to bring chemotherapy-related severe toxicity compared with radiotherapy alone. Firstly, MTT assay revealed that downregulating XPA expression in NPC HONE1 and CNE1 cells decreased IC_50_ of cisplatin and sensitized cells to cisplatin. XPA expression was detected by immunohistochemistry in cancer tissues from locally advanced NPC patients treated with platinum-based chemoradiotherapy. The relationships between XPA expression and clinicopathologic features, overall survival and progression-free survival of patients were evaluated. The results showed that XPA expression was not associated with clinicopathologic parameters, but was likely an independent prognostic factor for patient survival. High XPA level predicts a poor prognosis, and the prediction values were higher in subgroups of younger, higher EBV antibody titer, or treated with concurrent chemoradiotherapy. Combining XPA levels and T/N classifications, we successfully classified these patients into low, medium and high risk groups for platinum-based chemoradiotherapy. These findings suggest that XPA levels may be a potential predictor of prognosis in locally advanced NPC patients treated with platinum-based chemoradiotherapy, and helpful for selecting patients likely to need and benefit from this treatment in future.

## BACKGROUND

Nasopharyngeal carcinoma (NPC) has a high incidence in southern China [1, 2]. Platinum-based concurrent chemoradiotherapy is one of the standard treatment approaches for locally advanced NPC [3, 4]. However, meta analyses suggested that only 6% 5-year overall survival benefits were achieved in cisplatin-based concurrent chemoradiotherapy comparing with radiotherapy alone, whereas treatment-related death and severe acute toxicity obviously increased [5, 6]. Therefore, for better clinical outcome with less toxicity, it is very necessary to identify molecular markers which could predict the resistance of platinum-based chemotherapy.

In previous studies, we found that eukaryotic translation initiation factor 3a (eIF3a) confer to cispaltin sensitivity via downregulating the synthesis of nucleotide excision repair (NER) proteins, such as xeroderma pigmentosum complementation group A (XPA) and C (XPC), in NPC cell lines [7]. The overexpression of NER proteins theoretically promotes the activity of nucleotide excision repair, and consequently confers platinum resistance [8, 9]. Disappointedly, accumulating studies reveal discrepant results [10]. XPA has been reported to correlate with cisplatin cisplatin resistance in lung cancer cell lines [11, 12]. Down-regulating XPA expression or expressing a competitive, nonfunctional truncated XPA decreases the platinum resistance in lung cancer and prostate cancer DU15 cell lines, but not in prostate cancer PC3 cells [12-15]. Another reporter shows that overexpressing XPA does not increase the paltinum resistance in testis cancer 833K cells [16]. Several clinical researches have demonstrated that the expression of XPA proteins predicts improved outcome and good prognosis in ovarian carcinoma [17, 18] or no correlation with the response to cisplatin and overall survival in testicular germ cell tumors [19]. The role of XPA in response to cisplatin in NPC cell lines and NPC patients is not clear. In this study, we found that downregulating XPA sensitized NPC cells to cisplatin, and high expression of XPA was associated with poor prognosis in NPC patients treated with platinum-based chemoradiotherapy.

## RESULTS

### XPA contributes to cisplatin resistance in NPC cell lines

**Figure 1:**
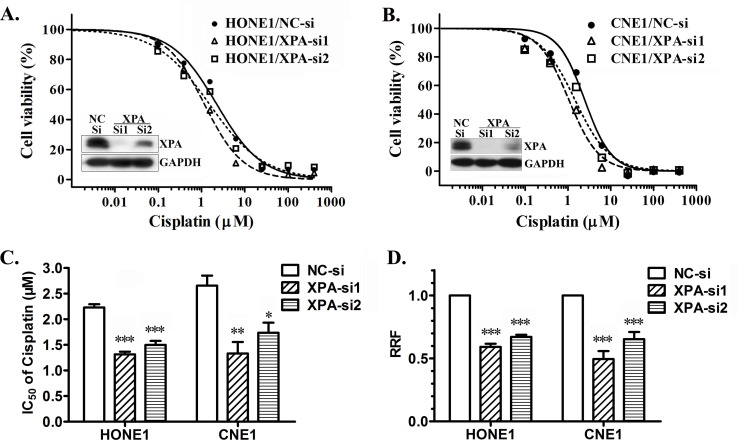
XPA contributes to cisplatin resistance in NPC cell lines IC_50_ values of cisplatin were measured by MTT assay after knocking down XPA expression in NPC cell line HONE1 and CNE1 cells by transient transfection of siRNAs (NC-si as negative control) for 24 h. **A.**, **B.** Representative dose-dependent cell viability curves in HONE1 and CNE1 cells (inner, Western blotting for XPA expression). **C.** Average IC_50_ values of cisplatin. **D.** The relative resistance factor (RRF). The data shown are from 4 independent experiments (*, *p* < 0.05; **, *p* < 0.01; ***, *p* < 0.001 compared with negative control).

To verify whether XPA is a cisplatin resistance factor in NPC cells, we firstly tested the half maximal inhibitory concentration (IC_50_) of cisplatin by MTT assay after knocking down XPA expression in NPC cell line HONE1 and CNE1 cells, or overexpressing XPA in CNE1 and CNE2 cells. AS shown in Figure 1A and 1B (inner), XPA is successfully knocked down by two siRNA oligonucleotides. Compared to cells transfected with negative control (NC) siRNA, all cells with reduced XPA expression are less resistant to cisplatin, the IC_50_ decreased 41.0% (XPA-si1) and 32.8% (XPA-si2) in HONE1 cells, and 49.8% (XPA-si1) and 34.6% (XPA-si2) in CNE1 cells respectively (Figure 1C); Consequently, the relative resistance factor (RRF) to cisplatin also decreased 40.9% (XPA-si1) and 32.9% (XPA-si2) in HONE1 cells, and 50.4% (XPA-si1) and 34.5% (XPA-si2) in CNE1 cells respectively (Figure 1D). In turn, the IC_50_ and RRF slightly increased in CNE1 cells (*p* < 0.05) and CNE2 cells (no significant) after ectopic XPA overexpression (Supplemental Figure S1). These results suggest XPA likely correlates with cisplatin resistance in NPC cell lines. Since NER pathway consists of at least other five factors except XPA, we speculate that the effect of XPA on NER activity may also be restricted by other factors, especially while overexpressing.

### Expression of XPA in NPC samples

**Figure 2:**
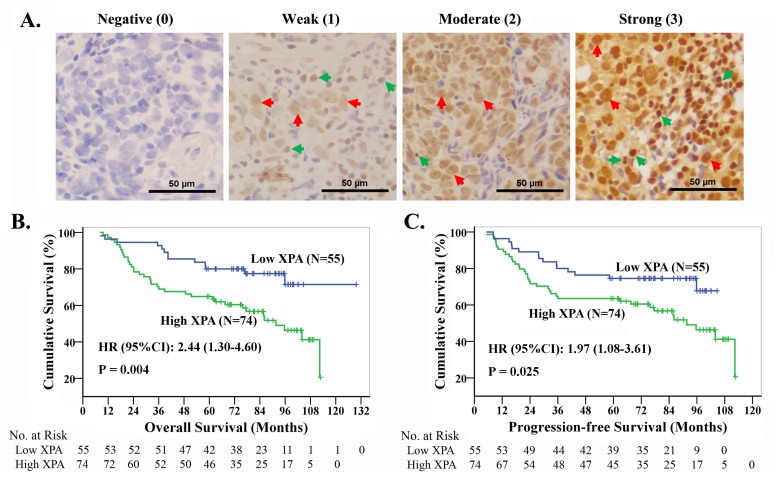
The high expression of XPA correlates with the prognosis in NPC patients treated with platinum-based chemoradiotherapy **A.** Representative pictures of immuohistochemistry for XPA. Brown staining, XPA positive; blue, cell nucleus. Red arrows point to XPA positive typical cancer cells, while green ones to XPA positive noncancerous cells. **B.**, **C.** Kaplan-Meier analysis and log-rank test for overall survival and progression-free survival according to XPA expression level.

Since XPA may correlates with cisplatin resistance in NPC cell lines, we wonder whether XPA level serves as a cisplatin resistance factor in NPC patients. So, we chose 129 newly diagnosed locally advanced NPC patients treated with radiotherapy plus induction chemotherapy or/and concurrent chemotherapy containing platinum-based regimens (at least 2 chemotherapy cycles) (Table 1), and tested XPA expression by immuohistochemistry (IHC) in biopsy tumor samples of these patients. Representative IHC images and the grades for XPA staining are shown in Figure 2A. XPA protein was localized in both nuclei and cytoplasm, but mainly in nuclei. XPA expression was observed high in lymphocyte and weak in nasopharyngeal epithelial cells (Figure 2A). As to tumor cells, XPA expression was detected in almost all samples (128/129), and high (H score ≥ 1.4) in 57.4% samples (Table 1).

**Table 1:** Baseline Characteristics of the Patients

Variable	No. of patients (%)
Gender	
Male	97 (75.2)
Female	32 (24.8)
Age	
≤ 50	82 (63.6)
>50	47 (36.4)
T classification	
T_1_	4 (3.1)
T_2_	26 (20.2)
T_3_	70 (54.3)
T_4_	29 (22.5)
N classification	
N_0_	14 (10.9)
N_1_	50 (38.8)
N_2_	60 (46.5)
N_3_	5 (3.9)
Clinical stage	
II	7 (5.4)
III	89 (69.0)
IVa/b	33 (25.6)
Histological type	
WHO type II	8 (6.2)
WHO type III	121 (93.8)
EBV VCA/IgA	
Negative	2 (1.6)
Positive ≤1:160	46 (36.5)
>1:160	78 (61.9)
EBV EA/IgA	
Negative	17 (13.5)
Positive ≤1:20	44 (34.9)
>1:20	65 (51.6)
Treatment strategy	
IC^a^ + RT^b^	70 (54.3)
CCRT^c^ +IC^a^	36 (27.9)
CCRT^c^ only	23 (17.8)
Vital status (at follow-up)	
Alive	79 (61.2)
Death	50 (38.8)
Expression of XPA	
Low expression	55 (42.6)
High expression	74 (57.4)

Note: a. IC, induction chemotherapy. b. RT, radiotherapy. c. CCRT, concurrent chemoradiotherapy.

### XPA expression was not associated with the clinicopathologic parameters

The relationships of XPA expression and the clinicopathologic parameters of NPC patients were analyzed by two-tailed χ^2^ test. The results showed that there were no significant correlations between XPA expression and all of the clinicopathologic parameters that we assessed, including gender, age (≤50, >50), T classification (T_1-2_, T_3-4_), N classification (N_0-1_, N_2-3_), clinical stage, histological type, VCA-IgA titer (≤1:160, >1:160), EA-IgA titer (≤1:20, >1:20) and treatment strategy (induction chemotherapy plus radiotherapy, concurrent chemoradiotherapy only/plus induction chemotherapy) (Table 2).

**Table 2:** Correlation between the clinicopathologic features and XPA expression

Characteristic	XPA^low^ (%)	XPA^high^ (%)	P value
Gender			
Male	40 (41.2)	57 (58.8)	0.681
Female	15 (46.9)	17 (53.1)	
Age (Medium age-yr)	46.0	47.0	0.716
≤ 50	36 (43.9)	46 (56.1)	
> 50	19 (40.4)	30 (59.6)	
T classification			
T_1-2_	16 (53.3)	14 (46.7)	0.209
T_3-4_	39 (39.4)	60 (60.6)	
N classification			
N_0-1_	24 (37.5)	40 (62.5)	0.287
N_2-3_	31 (47.79)	34 (52.3)	
Clinical stage			
II	4 (57.1)	3 (42.9)	0.547
III	39 (43.8)	50 (56.2)	
IVa/b	12 (36.4)	21 (63.6)	
Histological type			
WHO type II	3 (37.5)	5 (62.5)	1.000
WHO type III	52 (43.0)	69 (57.0)	
EBV VCA/IgA titer			
≤ 1:160	19 (39.6)	29 (60.4)	0.579
> 1:160	36 (46.2)	42 (53.8)	
EBV EA/IgA titer			
≤ 1:20	24 (39.3)	37 (60.7)	0.373
> 1:20	31 (47.7)	34 (52.3)	
Treatment strategy			
IC+RT^a^	35 (50.0)	35 (50.0)	0.076
CCRT^b^	20 (33.9)	39 (66.1)	

Note: a. IC+RT, induction chemotherapy plus radiotherapy. b. CCRT, including concurrent chemoradiotherapy only or plus induction chemotherapy.

### Survival analysis indicates that XPA expression is an independent prognostic factor

To investigate whether XPA expression serves as a prognostic marker in NPC patients treated with chemoradiotherapy containing platinum-based regimens, we performed univariate and multivariable Cox regression analysis. All variables were included (Table 2), except clinical stage (repeated with T and N classifications) and histological type (too less in type II). For univariate analysis, we found that T classification (Hazard ratio [HR] 2.54; 95% confidence interval [CI] 1.08-5.98; *p* = 0.027) and XPA expression (HR 2.44; 95%CI 1.30-4.60; *p* = 0.004) were significantly correlated with overall survival (OS), while only XPA expression (HR 1.97; 95%CI 1.08-3.61; *p* = 0.025) were significantly correlated with progression-free survival (PFS) (Supplemental Table S1). There were no significant correlations between survival and other parameters including gender, age, N classification, and treatment strategy. These results may due to a small sample pool.

However, multivariable analysis suggested XPA expression together with T and N classification were independent prognostic factors for OS and PFS (Table 3). Patients with T_3-4_ classification had significantly lower 5-year survival rates than those with T_1-2_ classification (OS, 67.7% *vs*. 83.3%; PFS, 65.7% *vs*. 76.7%); and 5-year survival rates were significantly lower in patients with N_2-3_ classification than those with N_0-1_ classification (OS, 64.6% *vs*. 78.1%; PFS, 58.5% *vs*. 78.1%). Kaplan-Meier analysis and log-rank test also indicated that high XPA level correlated significantly with poor OS and PFS, the cumulative 5-year survival rates were markedly lower in the high XPA group than those in the low XPA group (OS, 64.9% *vs*. 80.0%; PFS, 63.5% *vs*. 74.5%) (Figure 2B, 2C).

**Table 3:** Multivariate analysis of different prognostic variables in NPC patients by Cox regression analysis

Variable	OS		PFS	
	HR (95%CI)	*p* value	HR (95%CI)	*p* value
Gender (Male vs. Female)	0.91 (0.47-1.77)	0.777	0.83 (0.44-1.58)	0.567
Age (>50 vs. ≤50)	1.07 (0.58-1.95)	0.832	1.09 (0.61-1.96)	0.767
T classification (T_3-4_ vs. T_1-2_)	3.36 (1.28-8.84)	**0.014**	2.69 (1.10-6.56)	**0.029**
N classification (N_2-3_ vs. N_0-1_)	2.07 (1.12-3.83)	**0.020**	2.11 (1.16-3.85)	**0.015**
EBV VCA/IgA (>1:160 vs. ≤1:160)	1.29 (0.61-2.76)	0.504	1.24 (0.59-2.59)	0.566
EBV EA/IgA (>1:20 vs. ≤1:20)	0.63 (0.32-1.26)	0.194	0.74 (0.38-1.45)	0.381
Treatment strategy (CCRT^a^ vs. IC+RT^b^)	1.43 (0.81-2.55)	0.221	1.21 (0.69-2.15)	0.504
XPA expression (High vs. Low)	2.36 (1.22-4.60)	**0.011**	1.96 (1.05-3.68)	**0.035**

Note: a. IC+RT, induction chemotherapy plus radiotherapy. b. CCRT, including concurrent chemoradiotherapy only and induction chemotherapy plus concurrent chemoradiotherapy.

### Subgroup survival analysis

**Figure 3:**
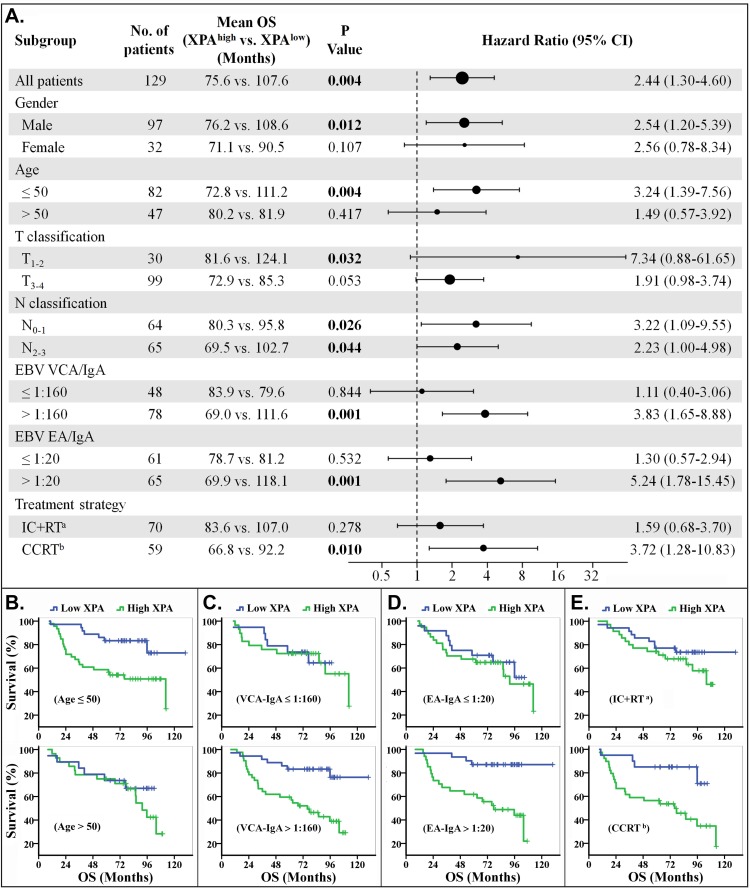
Stratified analysis for overall survival (OS) **A.** Hazard ratios for death are shown as a forest plot. The sizes of the circles are proportional to the number of events. **B.**-**E.** OS curves stratified by age **B.**, VCA-IgA **C.**, EA-IgA **D.** and treat strategy **E.**. (Note: a. IC+RT, induction chemotherapy plus radiotherapy. b. CCRT, including concurrent chemoradiotherapy only or plus induction chemotherapy).

**Figure 4:**
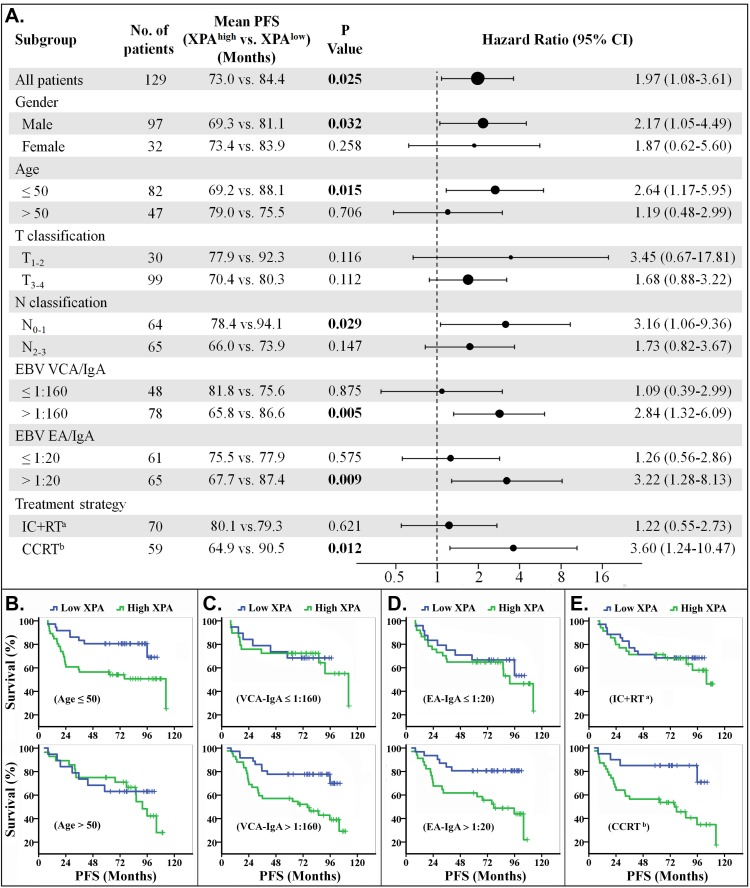
Stratified analysis for progression-free survival (PFS) **A.** Hazard ratios for disease progression are shown as a forest plot. The sizes of the circles are proportional to the number of events. **B.**-**E.** PFS curves stratified by age **B.**, VCA-IgA **C.**, EA-IgA **D.** and treat strategy **E.**. (Note: a. IC+RT, induction chemotherapy plus radiotherapy. b. CCRT, including concurrent chemoradiotherapy only or plus induction chemotherapy).

We also performed subgroup survival analysis to evaluate the value of XPA expression on prognostic prediction (OS and PFS) of patient subgroups stratified by gender, age, T classification, N classification, VCA-IgA, EA-IgA and treatment strategy. The results demonstrated that XPA expression possessed more prediction value on OS and PFS in the subgroups of younger patients (age≤50), VCA-IgA>1:160, EA-IgA>1:20, or treated with concurrent chemoradiotherapy only or plus induction chemotherapy. XPA expression also showed significant prognostic value (*p* < 0.05) in the subgroups of male, T_1-2_ classification and two N classifications (N_0-1_ and N_2-3_) for OS, in the ones of male and N_0-1_ classification for PFS (Figures 3 and 4, Supplemental Figures S2 and S3).

### Recursive partitioning analysis of survival

**Figure 5:**
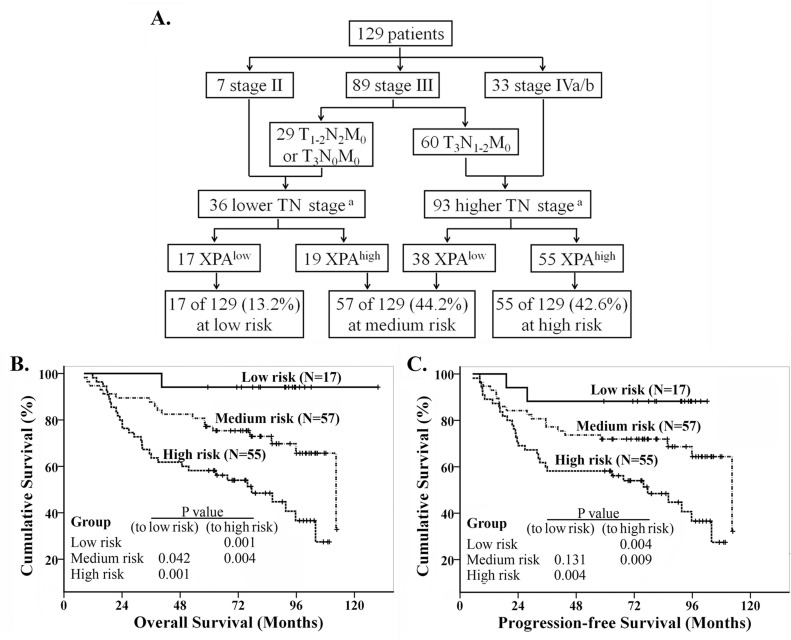
Recursive partitioning analysis of survival in all NPC patients **A.** Recursive partitioning analysis; **B.** Kaplan-Meier overall survival curves in the classified patients. **C.** Kaplan-Meier progression-free survival curves in the classified patients. Log rank analysis for *P* values. (Note: a. lower TN stage includes stage II, T_1-2_N_2_M_0_ and T_3_N_0_M_0_, higher TN stage includes T_3_N_1-2_M_0_ and stage IVa/b).

We investigated whether clinical stages, which combined T and N classifications together, affect the overall survival in this population of NPC patients, but the result was strange because no difference for OS between the patients at stage III and the ones at stage IVa/b (Supplemental Figure S4A). We wonder if one or several small populations in stage III patients had poor prognosis while others had good one. So we splitted stage III into two subgroups (IIIA, T_1-2_N_2_M_0_ or T_3_N_0_M_0_; IIIB: T_3_N_1-2_M_0_) (Figure 5A). Survival analysis revealed that there was significant difference for OS and PFS between IIIA and IIIB subgroups (Supplemental Figure S4B, S4C). The survival curve of stage IIIA patients was similar to the one of stage II, while that of stage IIIB was near to that of stage IVa/b (Supplemental Figure S4D). So we combined the stage IIIA and II to lower TN stage group, the stage IIIB and IVa/b to higher TN stage group (Figure 5A). We found great differences on OS and PFS between these two groups (Supplemental Figure S4E, S4F). Then, recursive partitioning analysis was performed to construct a decision tree for OS and PFS, using the significant independent prognostic factors including TN stage (lower/higher) and XPA expression. The results showed patients were classified into low, medium and high risk groups (Figure 5A), with 5-year OS (PFS) rates of 94.1% (88.2%), 77.2% (71.9%) and 58.2% (58.2%) respectively. Significant differences in survival were observed between the three groups, and the high risk group had significantly poorer prognosis compared to low and medium risk groups for OS and PFS (*p* < 0.01) (Figure 5B, 5C).

## DISCUSSION

Cisplatin and other platinum-based antitumor drugs have been exerted on a wide range of tumors including testicular, head and neck, lung, ovarian, bladder cancers [20, 21]. The major anticancer mechanism of platinum-based drugs is to form platinum-DNA adducts, a type of DNA lesion, by binding covalently to DNA strands, and cause a set of intracellular changes, and finally result in cellular apoptosis [22]. Although platinum has a miraculous initial therapeutic effect, unfortunately, it often leads to treatment failure because of intrinsic and extrinsic resistance to this drug. The potential mechanisms of platinum resistance [23] include reduced intracellular accumulation [24, 25] and increased detoxification of cisplatin [26], increased capacity of DNA damage repair [7, 9], inactivation of the apoptotic pathways [27] and other epigenetic alterations at molecular and cellular levels [28, 29]. Among of them, increased capacity of DNA damage repair, especially the nucleotide excision repair (NER), is proposed to be one of the most crucial determinants [9]. NER is a highly versatile and complicated pathway which could eliminate numerous types of DNA damage like caused by UV light and cisplatin [8]. This repair process could be generally divided into damage recognition, DNA opening, damage excision, and DNA resynthesis. It involves several factors assembling with an ordered and stepwise manner. Among of them, XPA binds to the damaged helical DNA strands and acts as a damage verifier [8]. So far, several studies have elucidated NER factors contribute to cisplatin resistance and hamper the effect of cisplatin-based treatment [30-33]. The NER factor XPC expression was reported to increase cisplatin resistance in lung adenocarcinoma cells and predict poor prognosis [30]. Excision repair cross-complementation group 1 is thought to confer cisplatin resistance in head and neck squamous cell carcinoma (HNSCC) cells and correlate with poor prognosis in HNSCC and locoregionally advanced NPC patients who receiving cispatin-based chemotherapy [31-33].

Although the critical role of XPA in NER and the important role of NER in cisplatin resistance have been accepted [8, 9], the relationship of XPA expression to cisplatin resistance and clinical prognosis of patients treated with platinum-based chemotherapy is still controversial according to previous reports [10-19]. A common XPA gene single nucleotide polymorphism (SNP), −4G/A in the fourth nucleotide before start codon, has been illuminated to be associated with susceptibility risk of lung cancer in Asian ethnicity [34], and has a significantly increased risk of progression and death in non-small cell lung cancer patients after radiotherapy and platinum-based chemotherapy [35].

Because of existing at the fourth nucleotide before start codon, this polymorphism will not affect protein structure and function. It is speculated that this polymorphism might affect the mRNA tertiary structure and stability or affect the binding between translational factors and mRNA, consequently interfere the translation of XPA. So we detected XPA at protein level, regardless of the polymorphism, to investigate the association of XPA and cisplatin resistance and clinical prognosis. In this study, we found that down-regulating XPA expression increased the cisplatin sensitivity in cultured NPC cells (Figure 1), so XPA is likely a cisplatin resistance factor. The investigation on clinical samples reveals that XPA expression is an independent prognostic factor and high XPA level is associated with poor prognosis in locally advanced NPC patients treated with radiotherapy plus platinum-based chemotherapy (Figure 2, Table 3). These findings suggest XPA level can be used to predict if a patient is likely sensitive or resistant to this treatment, and contribute to the development of individualized treatment regimens.

The treatment strategies included radiotherapy plus induction chemotherapy (IC+RT), concurrent chemoradiotherapy (CCRT) only or plus induction chemotherapy in our study. We found that the patients could be easily classified, by XPA expression level, into two groups with good or poor response to CCRT treatment regimen, but not to IC+RT regimen (Figures 3A, 3E, 4A and 4E). The phenomenon may result from the different cycles of platinum-base chemotherapy between CCRT treatment group and IC+RT group (4.53 cycles *vs*. 2.24 cycles). Subgroup survival analysis also demonstrated that XPA expression level had more prediction value on OS and PFS in the subgroups of EBV VCA-IgA>1:160 or EA-IgA>1:20, though EBV antibody titer alone wasn't correlative with prognosis in these 129 NPC patients (Figures 3A, 3C, 3D, 4A, 4C and 4D). Plasma EBV DNA has currently been considered as a biomarker for progression, relapse, and prognosis of nasopharyngeal carcinoma [36, 37]. These evidences indicate that EBV antibody titer might be a factor to improve the XPA-based prediction if a patient is likely resistant to platinum-base treatment. As to age classifications, XPA level displayed more high value in prognostic prediction for younger (age≤50) patients than the older ones (age>50) (Figures 3A, 3B, 4A and 4B). We assume that the confounding variables, such as complex influences resulted from senescence, attenuate the influence of platinum treatment in the elder. In addition, our results also showed XPA possess prediction value of survival in male subgroup but not in female (Figures 3A, 4A, Supplemental Figures S2A and S3A). This may due to a smaller population in female subgroup (only 32 patients). Considering that the hazard ratios of these two subgroups are very close (OS, 2.54 *vs*. 2.56; PFS, 2.17 *vs*. 1.87) (Figures 3A and 4A), we speculate that there might be no significant difference in prognostic prediction between male and female group.

In this study, we successfully classified locally advanced NPC patients into low, medium and high risk groups for the treatment regimen of radiotherapy combined with platinum-based chemotherapy, according to T, N classifications and XPA expression level (Figure 5). This classification may give a clue to physicians for making individualized treatment plans, or considering if the patients in high risk group should receive a different treatment. Previous reports have uncovered that there are many molecules and factors involved cispaltin resistance/sensitivity so far [7, 9, 14, 23-29]. We previously confirmed eIF3a confers cisplatin sensitivity via negatively regulating NER proteins *in vitro* study. In this study, we also detected eIF3a expression of NPC samples using IHC. However, the results showed that eIF3a expression isn't significantly correlative with either XPA level or patients' survival (data not shown). It seems that eIF3a expression is not suitable for predictive factor of prognosis in these NPC patients. For more precisely distinguishing patients, who might be resistant or sensitive to platinum-base treatment, more easily and accurately, we should perform a large scale of clinical trials to develop a comprehensive prediction model including more molecular factors and clinical parameters. Thus we can, hopefully, relieve those patients resistant to platinum-based chemotherapy from severe toxicity.

## MATERIALS AND METHODS

### Patients and tissue samples

One hundred and twenty-nine paraffin-embedded NPC tissue samples were collected from Sun Yat-sen University Cancer Center between 2002 and 2009. The cases eligibility criteria were as follows: histologically proven and newly diagnosed NPC without treatment history; locally advanced and no distant metastasis; no serious complications or other primary tumors; treated with chemoradiotherapy containing platinum-based regimens (at least 2 chemotherapy cycles) and regularly followed up in Sun Yat-sen University Cancer Center [38]. The study protocol was approved by the Institutional Review Board and Human Ethics Committee of Sun Yat-sen University Cancer Center, and informed consent was obtained from each patient. TNM staging was adjusted according to the AJCC Cancer Staging Manual (7^th^ edition). The median follow-up time was 76.1 months (rang, 8.2-129.7 months). The 5-year overall survival (OS) and progression-free survival (PFS) were 67.4% and 64.3%, respectively. Clinical information of the samples is summarized in Table 1.

### Materials

RPMI 1640, fetal bovine serum (FBS), TRIzol^®^ reagent, 3-(4,5-Dimethylthiazol-2-yl)-2,5-Diphenyltetrazolium Bromide (MTT), and the platinum SYBR Green qPCR SuperMix-UDG were purchased from Invitrogen (Carlsbad, CA, USA). M-MLV Reverse transcription system and GoTaq^®^ qPCR Master Mix were from Promega (Madison, WI, USA). BCA Protein Assay Kit was from KeyGEN Biotech (Nanjing, China). siTRAN transfection reagent was from OriGene (Beijing, China). Primers and small interfering RNAs (siRNAs) were synthesized respectively by Invitrogen (Shanghai, China) or GenePharma (Suzhou, China). Anti-XPA (sc-853), anti-GAPDH antibodies and HRP-conjugated secondary antibody were from Santa Cruz Biotechnology (Santa Cruz, CA, USA), ECL Western blotting detection reagents was from Amersham Biosciences (Piscataway, NJ, USA). Peroxidase Envision Kit was from Dako (Carpinteria, CA, USA). Cisplatin and all other reagents were of molecular biology grade and obtained from Sigma-Aldrich (Shanghai, China).

### Cells, siRNAs and transient transfection

Human NPC HONE1, CNE1 and CNE2 cells were cultured respectively in RPMI1640 medium containing 10% FBS and maintained in a humidified atmosphere with 5% CO_2_ at 37°C. For transient transfection, cells in 6-well plates were transfected with siRNAs or cDNA for XPA or negative control according to manufacturer's instruction. siRNAs sequences (Supplemental Table S2) referred to previously reports [12, 39].

### Real-time quantitative reverse transcriptase PCR (qRT-PCR)

qRT-PCR was conducted as described previously [7, 29]. Briefly, total RNA was extracted using TRIzol reagent. Two micrograms of total RNA was reverse transcribed to cDNA, sequentially subjected to qRT-PCR using primers shown in Supplemental Table S2. The threshold cycle (Ct) values were detected and normalized against that of β-actin internal control. The relative mRNA levels were calculated as the value of 2^ΔCt^ normalized to the control.

### Western blot analysis

Western blot analysis was performed as previously described [7, 29, 40]. Briefly, cell pellets were lysed and centrifuged to remove insoluble cell debris. The protein concentrations in supernatants were measured using BCA Protein Assay Kit, proteins were then separated by 10% SDS-PAGE followed by transfer to PVDF membranes. The blots were probed with anti-XPA (1:1,000) or anti-GAPDH (1:2,000) antibody, followed by reaction with HRP-conjugated secondary antibody. Signals were enhanced by ECL detection system and captured with X-ray film.

### Cell viability assay

Cell viability was determined by MTT assay. Briefly, cells were seeded in 96-well plates in a density of 2000 cells/well and incubated for 24 h followed by treatedcisplatin treatment for 72 h. Then cells were stained with MTT followed by determination of OD_570 nm_ with a reference wavelength at 630 nm. The data were analyzed using GraphPad Prism 5 software to obtain the IC_50_. The relative resistance factor (RRF) was calculated by dividing the IC_50_ value by that of control group.

### Immunohistochemistry

Immunohistochemistry was performed mainly as previously described [38, 41]. Briefly, tissues were fixed with formalin and embedded with paraffin then sectioned to a thickness of 4 μm. After routine deparaffinization, rehydration, blocking with hydrogen peroxide, sections were exposed to 10mM citrate buffer (PH 6.0) and heated at 95°C for 25 min in a water bath for antigen retrieval. Then the slides were incubated with anti-XPA antibody (1:400) overnight at 4°C, followed by incubation with HRP-conjugated secondary antibody and visualized by peroxidase Envision Kit. Sections were counterstained with hematoxylin.

The stained slides were evaluated independently by two pathologists who were blinded to clinical parameters. The staining intensity of cancer cells (excluding noncancerous cells, especially lymphocytes) were graded on a scale of 0 to 3 (I_0_, I_1-3_): negative staining (0), weak staining (1), moderate staining (2), and strong staining (3). The percentages of tumor cells in each grade (P_0_, P_1–3_) were recorded as 5% increments from 0 to 100% respectively. The final H scores were accumulated as follows formula: H score = I_1_×P_1_+ I_2_×P_2_ +I_3_×P_3_. ROC curve analysis was used to determine the cutoff value for XPA high expression group and low expression group. The H score that was closest to the point with both maximum sensitivity and specificity was selected as the cutoff value (H score = 1.40), and defined high XPA expression while H score ≥ 1.4 and low XPA expression while H score < 1.4.

### Statistical analysis

All statistical analyses were performed by using SPSS software package (version 16.0). The two-tailed χ^2^ test was used to assess the correlation of XPA expression with clinicopathological parameters. Kaplan-Meier and log-rank tests were used to analyze patient survival time and curves. Cox proportional model was used to calculate the multivariate hazard ratios for clinicopathological parameters and the XPA expression level with respect to overall survival (OS) and progression-free survival (PFS). The two-tailed t-test was used for comparisons of significance of the *in vitro* data. *P* values <0.05 were considered statistically significant.

## SUPPLEMENTARY MATERIAL FIGURES AND TABLES


