# Supplementary material for: High expression of XPA confers poor prognosis for nasopharyngeal carcinoma patients treated with platinum-based chemoradiotherapy
Source: Oncotarget. 2015 Jun 10;6(29):28478–90. doi: 10.18632/oncotarget.4424 (PMC4695073; doi:10.18632/oncotarget.4424)
Supplement: Supplementary file 1 [file oncotarget-06-28478-s001.pdf]

High expression of XPA confers poor prognosis for nasopharyngeal carcinoma patients treated with platinum-based chemoradiotherapy

Supplementary Material

Table S1: Univariate Cox regression analysis

**Variable**	**OS**		**PFS**	
	HR (95%CI)	*p* value	HR (95%CI)	*p* value
Gender (Male vs. Female)	/	0.769	/	0.614
Age (>50 vs. ≤50)	/	0.650	/	0.647
T classification (T_{3-4} vs. T_{1-2})	2.54 (1.08-5.98)	**0.027**	/	**0.058**
N classification (N_{2-3} vs. N_{0-1})	/	0.428	/	**0.213**
EBV VCA/IgA (>1:160 vs. ≤1:160)	/	0.705	/	0.485
EBV EA/IgA (>1:20 vs. ≤1:20)	/	0.446	/	0.735
Treatment strategy (CCRT [a] vs. IC+RT [b])	/	0.090	/	0.254
XPA expression (High vs. Low)	2.44 (1.30-4.60)	**0.004**	1.97 (1.08-3.61)	**0.025**

Note: a. IC+RT, induction chemotherapy plus radiotherapy. b. CCRT, including concurrent chemoradiotherapy only or plus induction chemotherapy.

Table S2: Primer, siRNAs sequence.

Targets	Name	Direction	Sequences
**Small interfering RNAs (siRNAs)**			
XPA	XPA-si1	Sense	5’-r(ACACAAGCUUAUAACCAAA)dTdT-3’
		Antisense	5'-r(UUUGGUUAUAAGCUUGUGU)dTdT-3'
	XPA-si2	Sense	5’-r(GUCAAGAAGCAUUAGAAGA)dTdT-3’
		Antisense	5'-r(UCUUCUAAUGCUUCUUGAC)dTdT-3'
Scramble	NC-si	Sense	5’-r(UUCUCCGAACGUGUCACGU)dTdT-3’
		Antisense	5’-r(ACGUGACACGUUCGGAGAA)dTdT-3’
**Primers for Quantitative PCR**			
XPA		Forward	5'- TCCACATCATTCACAATGGG -3'
		Reverse	5'- TGTCGGACTTCCTTTGCTTC -3'
ACTB		Forward	5'- TGGCACCCAGCACAATGAA-3'
		Reverse	5'- CTAAGTCATAGTCCGCCTAGAAGCA-3'

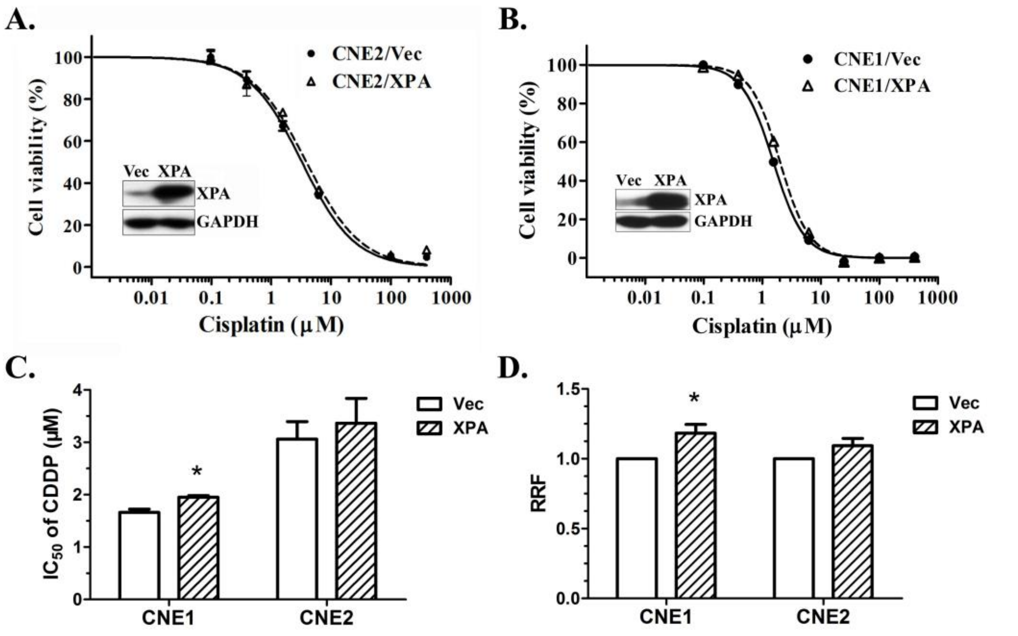

Figure S1: The effects of overexpressing XPA to cisplatin resistance in NPC cell lines. IC50 values of cisplatin were measured by MTT assay after overexpressing XPA in NPC cell line CNE2 and CNE1 cells by transient transfection of plasmids for 24 h. **A, B**: Representative dose-dependent cell viability curves in CNE2 (**A**) and CNE1 (**B**) cells (inner, Western blotting for XPA expression). **C**: Average IC_{50} values of cisplatin. **D**: The relative resistance factor (RRF). The data shown are from 4 independent experiments (*, $P < 0.05$ compared with control).

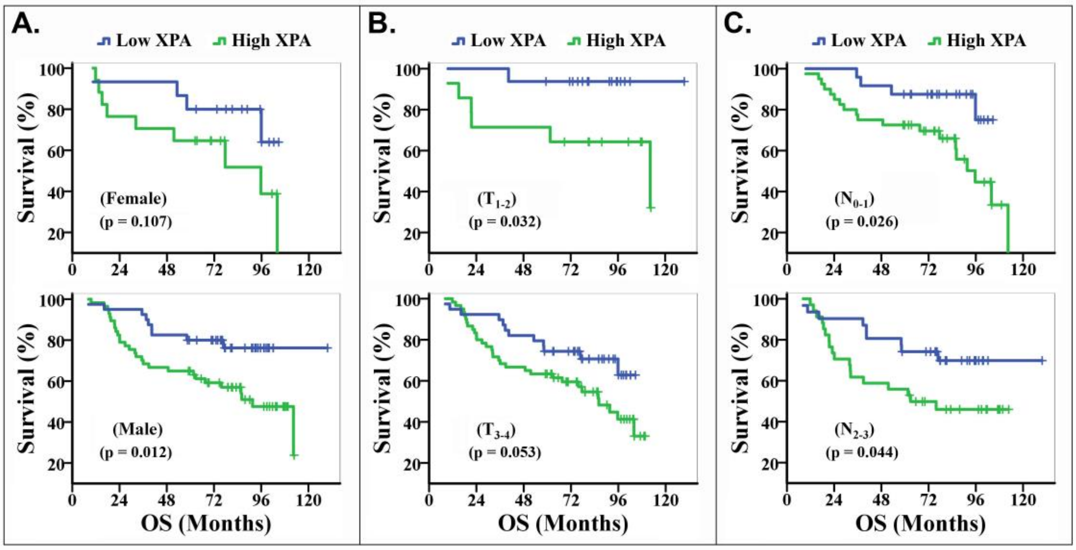

Figure S2: Stratified analysis for overall survival (OS). Subgroup OS analysis was performed after stratified by gender (**A**), T (**B**) and N classifications (**C**). **A:** OS curves for female or male group. **B**: OS curves for patients at T_{1-2} or T_{3-4} stage. **C**: OS curves for patients at N0-$_1$ or N_{2-3} stage. $p < 0.05$ indicates statistical significance.

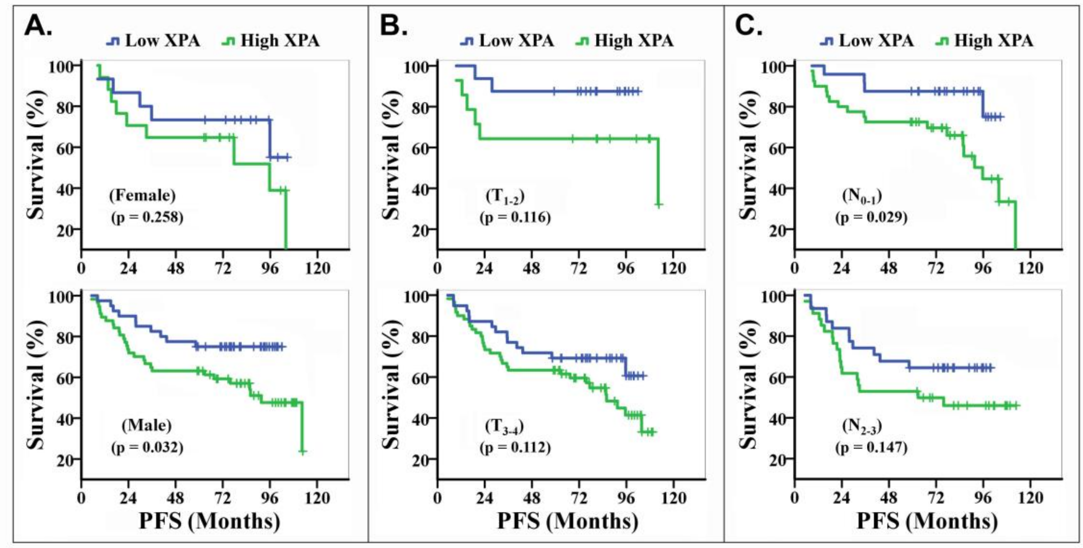

Figure S3: Stratified analysis for progression-free survival (PFS). Subgroup PFS analysis was performed after stratified by gender (**A**), T (**B**) and N classifications (**C**). **A:** PFS curves for female or male group. **B**: PFS curves for patients at T_{1-2} or T_{3-4} stage. **C**: PFS curves for patients at N0-$_{1}$ or N_{2-3} stage. $p < 0.05$ indicates statistical significance.

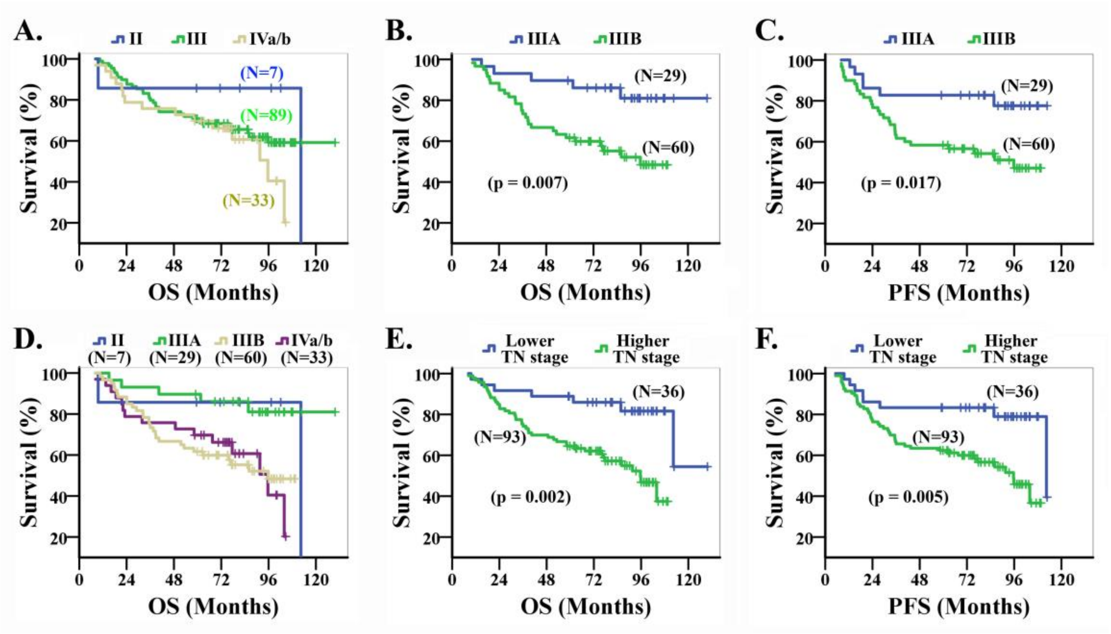

Figure S4: Kaplan-Meier analysis and log-rank test for overall survival (OS) or progression-free survival (PFS). A: OS curves for patients at stage II, III or IVa/b. **B, C**: OS and PFS curves for patients at stage IIIA ($T_{1-2}N_2M_0$ or $T_3N_0M_0$) or IIIB ($T_3N_{1-2}M_0$). **D**: OS curves for patients at stage II, IIIA, IIIB or IVa/b. **E, F**: OS and PFS curves for patients with lower TN stage (II, IIIA) or higher TN stage (IIIB or IVa/b).
